# Short PQ interval in alcohol withdrawal: diagnostic challenge in a psychiatric setting

**DOI:** 10.1192/j.eurpsy.2026.10834

**Published:** 2026-07-31

**Authors:** O. Plavkov

**Affiliations:** 19, Communal Nonprofit Enterprise “Vinnytsia Regional Clinical Psycho-Neurological Hospital Named After. Academician O. I. Yushchenko Of Vinnytsia Regional Council”, Vinnytsya, Ukraine

## Abstract

**Introduction:**

Chest pain in psychiatric inpatients often raises concern for acute coronary syndrome (ACS), particularly in those with alcohol use disorder, where somatic complaints and cardiological risks are common. Yet not all such pain reflects myocardial ischemia. Beyond the well-known “holiday heart syndrome,” rare conduction anomalies like Lown–Ganong–Levine (LGL) syndrome may appear with paroxysmal supraventricular tachycardia. Alcohol withdrawal, marked by autonomic overactivity, can further unmask these arrhythmias and complicate the clinical picture.

**Objectives:**

To demonstrate how alcohol withdrawal can mimic acute coronary syndrome and unmask rare conduction abnormalities such as LGL syndrome, emphasizing the importance of careful ECG interpretation in psychiatric settings.

**Methods:**

Clinical observation of a 49-year-old inpatient undergoing alcohol withdrawal. The patient was assessed through physical examination, continuous monitoring of vital signs, and standard 12-lead electrocardiography. Supportive care was provided within a psychiatric ward setting, with cardiological consultation available if symptoms worsened.

**Results:**

Electrocardiography revealed a short PQ interval (<120 ms), narrow QRS complexes, and no delta wave, consistent with a LGL conduction pattern. The episode was interpreted as a paroxysmal supraventricular tachycardia most likely triggered by autonomic flux during withdrawal. Symptoms subsided spontaneously with supportive care, without need for acute pharmacological or invasive intervention. An electrophysiological study would be required for definitive confirmation of the LGL syndrome.

This case illustrates how chest pain in psychiatric patients can easily be misinterpreted. In alcohol withdrawal, autonomic storm not only produces somatic sensations mimicking ACS but can also reveal pre-existing conduction anomalies. Misreading such ECG patterns as ischemic changes could unnecessarily prompt transfer to intensive care or invasive procedures. Previous reports in the literature have described arrhythmias unmasked by withdrawal, yet this remains under-recognized in psychiatric settings. Importantly, LGL syndrome may be overlooked because its ECG findings are subtle and may resemble normal variants. Differentiation from other pre-excitation syndromes, particularly Wolff–Parkinson–White, is essential to avoid misclassification. Careful ECG interpretation and awareness of such rare conditions can prevent misdiagnosis and reduce risk for the patient.

**Image 1:**
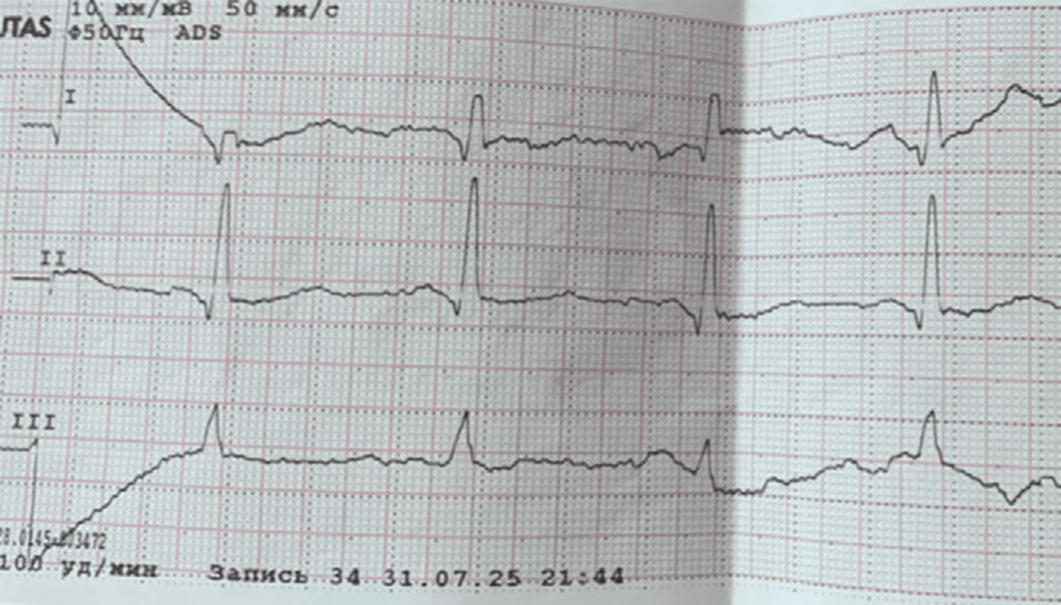
Image 1: Long description.

**Image 2:**
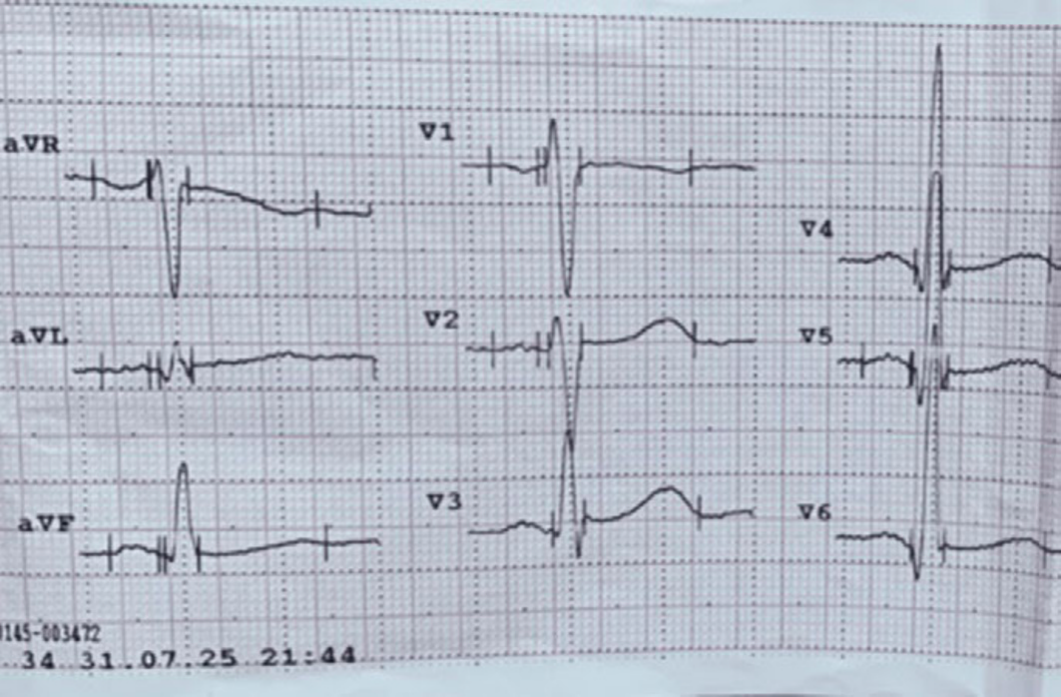
Image 2: Long description.

**Conclusions:**

Even the possible presence of conduction abnormalities must be considered in psychiatric contexts, both to guide monitoring and to avoid misdiagnosis and harm. This case underscores the need for collaboration between psychiatry and cardiology in the management of alcohol withdrawal, ensuring that uncommon but clinically relevant conduction anomalies are not missed.

**Disclosure of Interest:**

None Declared

## Long descriptions

### Image 1: Long description

A photograph of an E C G printout on pink grid paper.

Header Information:

At the top left, text reads 10 mm forward slash m V and 50 mm forward slash s. Below this are the initialisms J T A S, a symbol for 50 Hz, and A D S.

Waveform Panels:

- Lead I (Top): The trace begins with a significant downward baseline drift before stabilizing. It shows four distinct Q R S complexes. The first two complexes are followed by flat T-waves, while the final complex on the right shows an elevated, broad T-wave.

- Lead II (Middle): This trace is more stable, showing four sharp, upright R-waves. Each is preceded by a small P-wave and followed by a slightly irregular baseline.

- Lead III (Bottom): The trace starts with an upward baseline drift. It displays four Q R S complexes that are shorter in amplitude compared to Lead II. The baseline between complexes appears wavy, suggesting artifact or interference.

Footer Information:

At the bottom left, a numerical string 28.0145.3472 is visible above the text 100 beats per minute in Cyrillic (уд forward slash мин). A central footer note in Cyrillic reads ‘Record 34’ followed by the date and time 31 dot 07 dot 25 21 colon 44.

Navigate back to Image 1:.

### Image 2: Long description

The E C G is presented in a three-by-three grid on pink graph paper.

Left Column (Augmented Limb Leads):

- Top: a V R shows a small positive deflection followed by a deep negative Q R S complex and a downward sloping T wave.

- Middle: a V L shows a low-amplitude, biphasic Q R S complex with significant baseline noise.

- Bottom: a V F shows a small Q wave followed by a tall, narrow R wave and a flat T wave.

Middle Column (Precordial Leads V 1 to V 3):

- Top: V 1 shows a small R wave and a very deep S wave.

- Middle: V 2 shows a slightly taller R wave than V 1, a deep S wave, and a prominent upright T wave.

- Bottom: V 3 shows a tall R wave, a moderate S wave, and a prominent upright T wave.

Right Column (Precordial Leads V 4 to V 6):

- Top: V 4 shows a very tall, narrow R wave that extends nearly the full height of the panel.

- Middle: V 5 shows a tall R wave, though shorter than V 4, with a small S wave.

- Bottom: V 6 shows a moderate R wave and a small S wave.

At the bottom left, a timestamp reads 34 31 0 7 25 21 44.

Navigate back to Image 2:.

